# The Role of Detraining in Tendon Mechanobiology

**DOI:** 10.3389/fnagi.2016.00043

**Published:** 2016-02-29

**Authors:** Antonio Frizziero, Francesca Salamanna, Elena Della Bella, Filippo Vittadini, Giuseppe Gasparre, Nicolò Nicoli Aldini, Stefano Masiero, Milena Fini

**Affiliations:** ^1^Department of Physical and Rehabilitation Medicine, University of PaduaPadua, Italy; ^2^Laboratory of Biocompatibility, Technological Innovations and Advanced Therapies, RIT Department, Rizzoli Orthopedic InstituteBologna, Italy; ^3^Laboratory of Preclinical and Surgical Studies, Rizzoli Orthopedic InstituteBologna, Italy; ^4^Department of Experimental, Diagnostic and Specialty Medicine, University of BolognaBologna, Italy

**Keywords:** tendon, tenocyte, detraining, sudden detraining, systematic literature review

## Abstract

**Introduction**: Several conditions such as training, aging, estrogen deficiency and drugs could affect the biological and anatomo-physiological characteristics of the tendon. Additionally, recent preclinical and clinical studies examined the effect of detraining on tendon, showing alterations in its structure and morphology and in tenocyte mechanobiology. However, few data evaluated the importance that cessation of training might have on tendon. Basically, we do not fully understand how tendons react to a phase of training followed by sudden detraining. Therefore, within this review, we summarize the studies where tendon detraining was examined.

**Materials and Methods**: A descriptive systematic literature review was carried out by searching three databases (PubMed, Scopus and Web of Knowledge) on tendon detraining. Original articles in English from 2000 to 2015 were included. In addition, the search was extended to the reference lists of the selected articles. A public reference manager (www.mendeley.com) was adopted to remove duplicate articles.

**Results**: An initial literature search yielded 134 references (www.pubmed.org: 53; www.scopus.com: 11; www.webofknowledge.com: 70). Fifteen publications were extracted based on the title for further analysis by two independent reviewers. Abstracts and complete articles were after that reviewed to evaluate if they met inclusion criteria.

**Conclusions**: The revised literature comprised four clinical studies and an *in vitro* and three *in vivo* reports. Overall, the results showed that tendon structure and properties after detraining are compromised, with an alteration in the tissue structural organization and mechanical properties. Clinical studies usually showed a lesser extent of tendon alterations, probably because preclinical studies permit an in-depth evaluation of tendon modifications, which is hard to perform in human subjects. In conclusion, after a period of sudden detraining (e.g., after an injury), physical activity should be taken with caution, following a targeted rehabilitation program. However, further research should be performed to fully understand the effect of sudden detraining on tendons.

## Introduction

Tendons are a specialized tissues that join muscle to bone and are composed by extracellular collagen fibers arranged in regular arrays (Aslan et al., 2008). This mechanosensitive tissue shows detailed mechanical properties that allow it to adapt and respond to loading transmitted by muscles (Fang and Lake, 2015). This load transfer provide the principal mechanical stimulus for tendon cells (Kondratko-Mittnacht et al., 2015). These tensile loads are diverted to tendon cells through different matrix compartments and components. At cellular level, by various transmembrane structures and pathways, they are transduced from the exterior to intracellular biochemical responses (Kondratko-Mittnacht et al., 2015; Maeda and Ohashi, 2015).

While physiologic loads are required to maintain tendon homeostasis, (Galloway et al., 2013) unusual loading could direct to tendon injury, either through an acute traumatic injury or chronic, degenerative process (i.e., tendinopathy) resulting from an increase of microdamages and an altered cell/matrix response (Arnoczky et al., 2007; Magnusson et al., 2010). Histopathologicaly, tendinopathy is a unsuccessful healing response, represented by altered tenocytes proliferation, disruption and impaired organization of collagen fibers, increase in non collagenous matrix and neovascularization (Maffulli et al., 2011). In the chronic stage of tendinopathy, inflammation is absent or minimal, nevertheless it could play a role only in the initiation, but not in the propagation and progression, of the disease process (Maffulli et al., 2010). Even if tendinopathies also comprise conditions of damage to the tendon without symptoms, these pathologies frequently occur with pain in the injured tendon, which is accentuated or appears during palpation of the affected area or during active and passive movements involving the tendon (Franceschi et al., 2014). Tendon injury may not only lead in the lack of mobility or irregular joint kinematics, but could also result in damages to tissues adjacent to the joint. Muscle atrophy subsequent to tendon rupture is a frequent complication found by physicians and orthopedic surgeons. This condition proves significantly weaker musculature resulting in unfavorable functional consequences, with a consequent reduction in muscle force generation (Sandri, 2008; Zhang et al., 2013). Despite previous studies showed complete histological and biochemical characteristics of tendons rupture and some of these have been included into the clinical scenario, little is known concerning the mechanical response of muscles to tendon injury (Jamali et al., 2000; Derwin et al., 2006; Sandri, 2008; Charvet et al., 2012; Zhang et al., 2013). However, recently Zhang et al. (2013) demonstrated that tendon rupture has a supplementary influence on muscle biomechanics in comparison to disuse.

Due to their poor healing ability, tendon injuries represent an increasing problem in orthopedics as physicians are faced with a growing demand in sports and recreation and in the aging population (Kaux et al., 2011). Thus, primary disorders of tendons are a widely distributed clinical problem in society and hospital evidence and statistical data suggest that some tendons are more susceptible to pathology than others; these are the rotator cuff, Achilles tibialis posterior and patellar tendons. Although there are no specific figures in relation to tendon disease, several studies show that 16% of the population is affected from tendon pain (Urwin et al., 1998) and this rises to 21% when the statistics shift to elderly hospitals and community populations (Chard et al., 1991; Urwin et al., 1998). These numbers supplementary enhance in the sports community, in fact it was reported that 30–50% of all sporting injuries involve tendons (Kannus and Natri, 1997). Ordinarily, the major conditions affecting tendons are tendinitis and tendinosis; the first assumed to be accompanied by inflammation and pain, whereas the second can be caused by tendinous degeneration (Maffulli et al., 1998). It is assumed that these conditions are seldom spontaneous (Gibson, 1998) and are not caused by single factors. Rather, they are the end result of a variety of pathological processes (Riley, 2004; Rees et al., 2006) which can ultimately lead to the main clinical problem: loss of tissue integrity with full or partial tendon rupture.

Many intrinsic and extrinsic factors such as aging, gender, anatomical variants, obesity, systemic diseases, estrogen deficiency, drugs, sporting activities, physical loading, occupation, and environmental conditions could affect the biological and anatomo-physiological characteristics of the tendon (Nakama et al., 2005; Holmes and Lin, 2006; Torricelli et al., 2006, 2013; Frey and Zamora, 2007; Franchi et al., 2013; Frizziero et al., 2013, 2014; Malliaras et al., 2013; Moerch et al., 2013; Abate, 2014; Berardi et al., 2014; Boivin et al., 2014; Galdiero et al., 2014; Hast et al., 2014; Oliva et al., 2014a,b; Snedeker and Gautieri, 2014; Sandberg et al., 2015). Thus, over the past decade, tendon and tenocyte adaptations in relation to immobilization, training, aging and medications have been the center of an growing number of studies (Maffulli et al., 2003; Sharma and Maffulli, 2005; Torricelli et al., 2006, 2013; Stanley et al., 2008).

**Figure 1:**
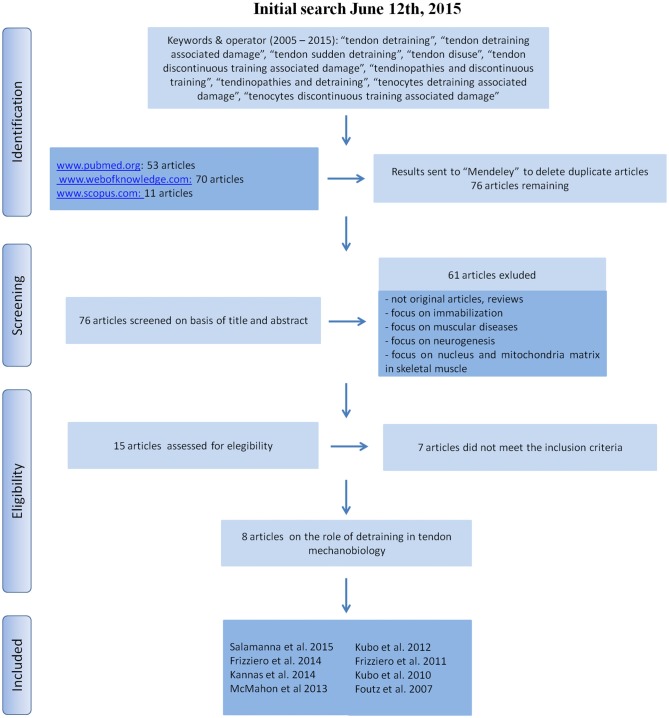
**Literature search strategy and criteria**.

While proper mechanical loads at physiological levels are typically helpful to tendons in terms of enhancing its mechanical properties, recent preclinical and clinical studies examining the effect of detraining on tendon, showed alterations in its structure and morphology and in tenocyte mechanobiology. However, there is a paucity of data that evaluated the impact that detraining may have on tendon. Thus, it has not yet been understood how tendons behave to a period of training followed by cessation of training. Nevertheless, to guide rehabilitation and/or athletic programs it is necessary to elucidate tendon adaptation after sudden detraining. Therefore, within this descriptive systematic literature review, we summarize the studies where tendon detraining was examined.

## Materials and Methods

### Descriptive Literature Review

According to the Preferred Reporting Items for Systematic Reviews and Meta-Analyses (PRISMA) a systematic search was carried out for this descriptive literature review (see Figure 1 for details) in three databases (www.pubmed.org, www.webofknowledge.com, www.scopus.com). The keywords were “tendon detraining”, “tendon detraining associated damage”, “tendon sudden detraining”, “tendon disuse”, “tendon discontinuous training associated damage”, “tendinopathies and discontinuous training”, “tendinopathies and detraining”, “tenocytes detraining associated damage”, “tenocytes discontinuous training associated damage”. We sought to identify studies in which tendon detraining was examined. Publications from 2005 to 2015 (original articles in English) were included. The reference lists from the articles included in this review were analyzed to recognize additional studies that were not found by the initial search. A public reference manager (www.mendeley.com) was used to delete duplicate articles.

## Results

An initial literature search yielded 134 references. Fifty-three articles were identified using www.pubmed.org, 70 articles using www.webofknowledge.com and 11 articles were found in www.scopus.com. The resulting references were submitted to a public reference manager (Mendeley 1.13.8, www.mendeley.com) to delete duplicate articles. Of the 76 remaining articles, 15 publications were extracted based on the title for further analysis. Abstracts and whole articles were then reviewed to ascertain whether the publication met the inclusion criteria and eight articles (four preclinical studies, one *in vitro* and three *in vivo*, and four clinical studies) were considered appropriate for the review (Figure 1). From the reference lists of the included articles, no supplementary publications were identified. We did not perform meta-analyses of the selected studies, but quoted the results in a descriptive fashion.

### Preclinical Studies

This revised literature comprised four preclinical studies, an *in vitro* and three *in vivo* reports, respectively on tenocytes from patellar tendon (Salamanna et al., 2015) and on patellar (Frizziero et al., 2011, 2015) and gastrocnemius (Foutz et al., 2007) tendon of detrained animals (Table 1). Concerning the *in vitro* study patellar tendon tenocytes from rats subjected to training and to sudden detraining were examined. Rats were trained for 10 weeks on a treadmill (speed of about 25 m/min, corresponding to ~65–70% VO_2_max) and successively caged without exercise for further 4 weeks. Tenocytes from patellar tendon were cultured to evaluate morphology, viability, proliferation and metabolic activity****. It was found that detraining in the short-term alters tenocyte synthetic and metabolic activity (C-terminal-propeptide of type I collagen, collagen III, fibronectin, aggrecan, tenascin-c, interleukin-1β, matrix-metalloproteinase-1 and -3). These results indicated that tenocytes do not merely have a passive role but play an important function during detraining (Salamanna et al., 2015). Similarly results were found by the same authors also when the patellar tendons of detrained rats were studied by histology and histomorphometry (Frizziero et al., 2011, 2015). In fact, the studies showed alteration in tendon morphology and also in its enthesis due to discontinuation of training. These alteration involved proteoglycan content, collagen fiber organization with an increase of collagen III and a decrease of collagen I, which means less resistance to stress, and a related increased risk of rupture. Differently from the above mentioned studies, Foutz et al. (2007) investigated the mechanical adaptability responses due to disuse on the biomechanical properties of the gastrocnemius tendon of chicks. Chicks were trained for 3 weeks on a treadmill (speed of 0.22 m/s, for 5 min) and successively immobilized in a whole body suspension system for further 2 weeks. It was found that structural strength and toughness of the gastrocnemius tendon were reduced by 10 and 30%, respectively, whereas the material strength, material toughness, and material stiffness of the tendon increased by approximately 75, 65, and 70%, respectively. These results showed that the chicken gastrocnemius tendon reacts to mechanical disuse as foretold by the mechanobiology process (Foutz et al., 2007).

**Table 1:** **Preclinical studies on role of detraining in tendon mechanobiology**.

Experimental	Type of tendon	Control group	Training protocol	Detraining protocol	Analysis	Main results	Referenceset-up
*In vitro* model	Rat patellar tendon tenocyte	Untrained patellar tendon tenocyte Trained patellar tendon tenocyte	10 week on a treadmill (~65–70% VO_2_max)	Caged without exercise for 4 weeks	Transmission- electronic- microscopy, C-terminal- propeptide of type I collagen, collagen III, fibronectin, aggrecan, tenascin-c, interleukin-1β, matrix- metalloproteinase-1 and-3	Altered tenocyte synthetic and metabolic activity	Salamanna et al. (2015)
*In vivo* model	Chicken gastrocnemius tendon	No control group	3 week on a treadmill (speed of 0.22 m/s, for 5 min)	Controls or immobilized for 2 weeks	Tendon midregion cross-sectional area and biomechanical properties	Gastrocnemius tendon responds to mechanical disuse as predicted by the mechanobiology process	Foutz et al. (2007)
*In vivo* model	Rat patellar tendon	Untrained patellar tendon Trained patellar tendon	10 week on a treadmill (~60% VO_2_max)	Caged without exercise for 4 weeks	Collagen fiber organization and proteoglycan content	Low proteoglycan content and collagen fiber organization	Frizziero et al. (2011)
*In vivo* model	Rat patellar tendon	Untrained patellar tendon Trained patellar tendon	10 week on a treadmill (~65–70% VO_2_max)	Caged without exercise for 4 weeks	Structure and morphology (modified Movin score, tear density, collagen type I and III)	Altered structure and morphology with the highest Movin score values, the highest percentage of collagen III and the lowest of collagen I	Frizziero et al. (2015)

### Clinical Studies

The PubMed, Web of Knowledge and Scopus search strategy identified four clinical papers that examined the impact that detraining may have on tendons (Table 2). Several studies showed that tendon characteristics influence the performances during stretch-shortening cycle exercises (Bojsen-Møller et al., 2005; Kubo et al., 2007; Stafilidis and Arampatzis, 2007); thus, information on the time course of changes in tendon characteristics during training and detraining is critical for the progress of performances in the athletic field. To evaluate the time course of modifications in mechanical and morphological properties of tendon during detraining, Kubo et al. (2010) examined these variables in eight volunteered men that executed unilateral knee extension exercise in a seated position. Subjects were trained 4 times per weeks for 3 months and detrained for the following 3 months. Results of this study showed that tendon stiffness was significantly increased after 3 months of training, while the maximal elongation was unaltered. Conversely, during the detraining period, tendon showed greater values of maximal elongation compared to the post-training, and tendon stiffness decreased to the pre-training levels after 2 months of detraining (Kubo et al., 2010). With a similar methodology, the same authors in 2012 focused more specifically on the alterations found in the human Achilles tendon during training and detraining (Kubo et al., 2012). In addition, they measured the blood volume and oxygen saturation of tendon, and evaluated the serum concentrations of markers of collagen type I synthesis. Results were similar to the previous study ones: the elongation values did not change after training but increased significantly during detraining; tendon stiffness increased only after 3 months of training and rapidly decreased during detraining. Thus, authors showed that during detraining, the sudden decrease in tendon stiffness might be linked to modifications in the structure of collagen fibers within the tendon. In addition, no significant alterations in blood supply or collagen synthesis were observed (excluding an increase in procollagen peptides after 2 months of training; Kubo et al., 2012).

**Table 2:** **Clinical studies on role of detraining in tendon mechanobiology**.

Type of tendon	Patients	Training protocol	Detraining protocol	Analysis	Main results	Reference
Patellar tendon	8 (training group); 6 (control group)	Unilateral isometric knee extension, 4 times/week, 3 months	Return to usual levels of physical activity, 3 months	- Tendon elongation by ultrasounds; - Cross-sectional areaby MRI	Greater values of tendon elongation, decrease in tendon stiffness during detraining	Kubo et al. (2010)
Achilles tendon	9 (training group); 7 (control group)	Unilateral (left side) isometric plantar flexion exercise, 4 times/week, 3 months	Return to usual levels of physical activity, 3 months	- Tendon elongation by ultrasounds; - Cross-sectional areaby MRI; - Blood supply and oxygen saturation; - Serum concentration of BAP and P1P by ELISA	Tendon elongation increased and stiffness rapidly decreased after detraining	Kubo et al. (2012)
Patellar tendon	10 (training with the MTC at a shortened position); 11 (MTC at a lengthened position); 11 (wide range of motion); 10 (control group)	Resistance training, 3 times/week 8 weeks	4 weeks of detraining	- Patella moment arm by DEXA; - Tendon elongation and stiffness by ultrasounds; - Circulating TGF-β1 levels by ELISA	No significant alterations in patella tendon dimensions or circulating TGF-β1 levels following training or detraining in any of the groups	McMahon et al. (2013)
Achilles tendon	10 (training on inclined ground); 10 (training on plain ground)	Plyometric training	4 weeks of detraining	Aponeurosis strain of MG	Strain was decreased from 22.7% (±0.05) to 16.3% (±0.05) after detraining period	Kannas et al. (2015)

Recently McMahon et al. (2013) evaluated the patella tendon properties during detraining (1 month), after a 3 months period of training with different strains. The patella moment arm, the perpendicular distance between the tibiofemoral contact point and the mid-portion of the tendon, was estimated using dual-energy x-ray absorptiometry (DEXA) scan images. Tendon elongation and stiffness were measured by ultrasonic analyses and tendon forces were calculated as the ratio between the measured torque and the patella moment arm. Furthermore, they evaluated the circulating transforming growth factor (TGF)-β1 levels as it is associated to exercise-induced response to mechanical loading of muscle and tendon. The authors found no significant alterations in patella tendon dimensions or circulating TGF-β1 levels following training or detraining. However, the training groups with the muscle-tendon complex at a lengthened position or over a wide range of motion better maintained adaptations compared to the training in a shortened position subsequent to detraining, with a pattern of slower loss of progress at the early phase of detraining in all training groups.

Finally Kannas et al. (2015) analyzed the effect of 4 weeks of detraining on the mechanical properties of medial gastrocnemius aponeurosis into two groups that performed plyometric training on incline and plane ground. They evaluated the aponeurosis strain of medial gastrocnemius and found that it decreased after detraining; the ankle muscle tendon complex properties withdrew to the pre-training values with lower performances. These findings suggested that after 4 weeks of detraining, ankle muscle tendon complex properties withdraw to the pre-training values with lower performance (Kannas et al., 2015).

## Discussion

The tendon is a connective tissue responsible for the transmission of force from the muscular tissue to the bones, promoting body movement. It is not a static tissue, preferentially it adapts itself in compliance to the level, direction and frequency of the load that is applied to it with a process of remodeling possibly executed by tenocytes.

It was shown that appropriate mechanical loads are useful to tendons by improving their anabolic processes and it is undertaken or prescribed for different reasons such as sports performance, general health, functional maintenance, recovery (e.g., following injury, illness/diseased states) and also to compensate the effects of ageing. However, extreme mechanical loads are harmful to tendons by bringing catabolic processes such as matrix degradation. Immobilization or disuse of tendons also leads catabolic effects on it. Differently there are few data that examined the impact that detraining may have on tendons. Thus, the present descriptive systematic literature review tried to summarize the effects of discontinuing physical activity on tenocyte metabolism and/or in tendon morphology in order to elucidate the mechanism behind these changes.

All examined studies, both preclinical and clinical, observed that discontinuing activity negatively influence tendon structure and morphology, albeit with differences in the training and/or detraining protocols, in the types of tendons, in subjects involved, in the study design or in the experimental setting involved. The results of all these studies suggested that after a period of sudden detraining (such as after an injury) physical activity should be restarted with caution and with appropriate rehabilitation programs because cessation of activity causes modifications in tenocytes and tendons metabolism, morphology, i.e., in collagen type I and III synthesis, collagen organization, cellularity, vascularity, proteoglycan content, tear density, mechanical properties.

Notwithstanding the alterations highlighted in the reviewed articles after tendon detraining, some limitations of the examined studies should be also considered. In fact, this systematic review has as its main focus not only to bring together major works involving major changes in morphological and structural properties of tendons during detraining, but also to examine the methodological process on which the articles were based to assess the trustworthiness of the results found.

In relation to the results obtained in the *in vitro* study examined in this review (Salamanna et al., 2015), that showed a decrease of tendon mitochondrial area, rough endoplasmic reticulum area, C-terminal propeptide of type I collagen, fibronectin, aggrecan and tenascin-c synthesis and presence of inflammatory cytokine production, we have to consider that tenocytes from animals subjected to sudden detraining were studied. In addition, results were obtained in *in vitro* cultured cells, which were not any longer structured into tissues, but in monolayer and static conditions. Thus, it is probable that the performance of explanted tendon cells is not equal to the performance of tendon cells in their native matrix environment *in vivo* (Fu et al., 2008; Leigh et al., 2008). However, these results indicated that the tendon does not operate as a inert connector between muscles and bone, but dynamically responds to mechanical loading.

The three preclinical studies examined in this review employed a rat or chicken animal model that may not be fully representative of human conditions but the invasive analyses conducted in these studies permitted a depth investigation for the advancement of knowledge of many aspects on tendon response to detraining (Foutz et al., 2007; Frizziero et al., 2011, 2015). Moreover, looking at the literature, rat and rodents are the most used animals when mechanical load with treadmill running is used (Warden, 2009; Lui et al., 2011). In fact, the results of these *in vivo* studies demonstrated that the adopted running protocol did not induce tendinopathy or other pathologic changes in hindlimbs. Another methodological process that must be considered is that in these studies all morphometric parameters were measured by 2D image analysis, while other investigation methods, such as micro-MRI, may allow a more in-depth understanding of tendon structure. However, as for the reviewed *in vitro* paper, these *in vivo* results provide interesting data for both sports medicine practitioners and orthopedic surgeons, wishing to prevent the pathological or degenerative modification that affect these structures.

Great variability was noted in the four clinical studies (Kubo et al., 2010, 2012; McMahon et al., 2013; Kannas et al., 2015) that analyzed the effects of detraining. In fact, these studies involved different tendons (Achilles, gastrocnemius, patellar), different types of exercise (isometric knee extention, resistance training, plyometric training on incline and plane ground), different training and detraining periods (3 and 4 months) and different types of analyses (Dual Energy X-Ray Absorptiometry, ultrasonography, electromyography). Furthermore, it is important to point out that the different effects of detraining on tendons depends not only on the above mentioned variables, but also on the patient intrinsic characteristics, that are affected by age, gender, drug assumption, the presence of systemic or genetic or endocrine diseases (i.e., obesity, diabetes, Cushing syndrome, hypercholesterolemia, osteoporosis). In fact, recently it was shown that proliferation and synthetic activity of tenocytes are negatively affected by aging and estrogen deficiency (Torricelli et al., 2013). In addition, clinical studies did not permit a depth understanding of the alteration in tendon metabolism and morphology (i.e., expression of type I collagen, fibronectin, aggrecan and tenascin-c synthesis and/or presence of inflammatory cytokine, cellularity, vascularization, fibers arrangements etc.). However, despite these limitations these clinical studies indicate that tendons may be susceptible to detraining. These findings could have a direct relevance to functional rehabilitation practices showing that after a period of sudden detraining, physical activity should be restarted with caution.

Despite the fact that the examined studies showed a potential negative effect of detraining on tenocytes and tendons, there is a paucity of preclinical and clinical studies that examined the importance that cessation of training may have on tendon. These results should be confirmed by other preclinical and clinical research in order to completely comprehend the effect of detraining on tendons. In particular, several aspects should be further studied and refined in order to improve our understanding on the role of detraining in tenocytes and tendon mechanobiology: (1) standardization of the training and detraining protocols in both preclinical and clinical research; (2) development of systems that reproduce tendon detraining in culture with high reliability to native tendon; (3) comprehend how tenocytes respond to detraining and how they mechano-regulate their response; (4) evaluate the presence of altered tendon structure and/or morphology due to detraining in its various stages; and (5) evaluation of the role of other tissues (bone, muscle, nerve, vascularity, etc.) on tendon mechanobiology during detraining. Finally an integrated, collaborative multi-disciplinary multiscale approach is likely to yield the greatest advances in this field.

## Author Contributions

AF: has conceived the study and was involved in drafting the manuscript, FS: has conceived the study and was involved in drafting the manuscript, EDB: was involved in the literature search and in the data analysis, FV: was involved in the literature search, GG: was involved in drafting the manuscript, NNA: was involved in the literature search, SM: participated in drafting the manuscript and in the data analysis, MF: has conceived the study and was involved in drafting the manuscript.

## Acknowledgments

This work was supported by grants from Rizzoli Orthopaedic Institute (Ricerca Corrente) and by the Operational Program ERDF 2007-2013 of region Emilia-Romagna: Activity 1.1 “Creation of technology centers for Industrial research and technological transfer”.

## Conflict of Interest Statement

The authors declare that the research was conducted in the absence of any commercial or financial relationships that could be construed as a potential conflict of interest. The handling Editor declared a shared affiliation, though no other collaboration, with several of the authors (AF, FV, GG, SM) and states that the process nevertheless met the standards of a fair and objective review.

## References

[B1] Abate M. (2014). How obesity modifies tendons (implications for athletic activities). Muscles Ligaments Tendons J. 4, 298–302. 10.11138/mltj/2014.4.3.298 25489546 PMC4241419

[B2] Arnoczky S. P. Lavagnino M. Egerbacher M. (2007). The mechanobiological aetiopathogenesis of tendinopathy: is it the over-stimulation or the under-stimulation of tendon cells? Int. J. Exp. Pathol. 88, 217–226. 10.1111/j.1365-2613.2007.00548.x 17696902 PMC2517314

[B3] Aslan H. Kimelman-Bleich N. Pelled G. Gazit D. (2008). Molecular targets for tendon neoformation. J. Clin. Invest. 118, 439–444. 10.1172/JCI33944 18246194 PMC2214706

[B4] Berardi A. C. Oliva F. Berardocco M. la Rovere M. Accorsi P. Maffulli N. (2014). Thyroid hormones increase collagen I and cartilage oligomeric matrix protein (COMP) expression *in vitro* human tenocytes. Muscles Ligaments Tendons J. 4, 285–291. 10.11138/mltj/2014.4.3.285 25489544 PMC4241417

[B5] Boivin G. P. Elenes E. Y. Schultze A. K. Chodavarapu H. Hunter S. A. Elased K. M. (2014). Biomechanical properties and histology of db/db diabetic mouse achilles tendon. Muscles Ligaments Tendons J. 4, 280–284. 10.11138/mltj/2014.4.3.280 25489543 PMC4241416

[B6] Bojsen-Møller J. Magnusson S. P. Rasmussen L. R. Kjaer M. Aagaard P. (2005). Muscle performance during maximal isometric and dynamic contractions is influenced by the stiffness of the tendinous structures. J. Appl. Physiol. (1985) 99, 986–994. 10.1152/japplphysiol.01305.2004 15860680

[B7] Chard M. D. Hazleman R. Hazleman B. L. King R. H. Reiss B. B. (1991). Shoulder disorders in the elderly: a community survey. Arthritis Rheum. 34, 766–769. 10.1002/art.1780340619 2053923

[B8] Charvet B. Ruggiero F. Le Guellec D. (2012). The development of the myotendinous junction. A review. Muscles Ligaments Tendons J. 2, 53–63. 23738275 PMC3666507

[B9] Derwin K. A. Baker A. R. Spragg R. K. Leigh D. R. Iannotti J. P. (2006). Commercial extracellular matrix scaffolds for rotator cuff tendon repair. Biomechanical, biochemical and cellular properties. J. Bone Joint Surg. Am. 88, 2665–2672. 10.2106/jbjs.e.01307 17142417

[B48] Fang F. Lake S. P. (2015). Multiscale strain analysis of tendon subjected to shear and compression demonstrates strain attenuation, fiber sliding and reorganization. J. Orthop. Res. 33, 1704–1712. 10.1002/jor.22955 26036894

[B10] Foutz T. Ratterman A. Halper J. (2007). Effects of immobilization on the biomechanical properties of the broiler tibia and gastrocnemius tendon. Poult. Sci. 86, 931–936. 10.1093/ps/86.5.931 17435028

[B11] Franceschi F. Papalia R. Paciotti M. Franceschetti E. Di Martino A. Maffulli N.. (2014). Obesity as a risk factor for tendinopathy: a systematic review. Int. J. Endocrinol. 2014:670262. 10.1155/2014/670262 25214839 PMC4156974

[B12] Franchi M. Torricelli P. Giavaresi G. Fini M. (2013). Role of moderate exercising on Achilles tendon collagen crimping patterns and proteoglycans. Connect Tissue Res. 54, 267–274. 10.3109/03008207.2013.807808 23758268

[B13] Frey C. Zamora J. (2007). The effects of obesity on orthopaedic foot and ankle pathology. Foot Ankle Int. 28, 996–999. 10.3113/fai.2007.0996 17880874

[B14] Frizziero A. Bonsangue V. Trevisan M. Ames P. R. Masiero S. (2013). Foot tendinopathies in rheumatic diseases: etiopathogenesis, clinical manifestations and therapeutic options. Clin. Rheumatol. 32, 547–555. 10.1007/s10067-012-2158-2 23274757

[B15] Frizziero A. Fini M. Salamanna F. Veicsteinas A. Maffulli N. Marini M. (2011). Effect of training and sudden detraining on the patellar tendon and its enthesis in rats. BMC Musculoskelet. Disord. 12:20. 10.1186/1471-2474-12-20 21247475 PMC3038990

[B16] Frizziero A. Salamanna F. Giavaresi G. Ferrari A. Martini L. Marini M.. (2015). Hyaluronic acid injections protect patellar tendon from detraining-associated damage. Histol. Histopathol. 30, 1079–1088. 10.14670/HH-11-605 25767092

[B17] Frizziero A. Vittadini F. Gasparre G. Masiero S. (2014). Impact of oestrogen deficiency and aging on tendon: concise review. Muscles Ligaments Tendons J. 4, 324–328. 10.11138/mltj/2014.4.3.324 25489550 PMC4241423

[B18] Fu S. C. Shum W. T. Hung L. K. Wong M. W. Qin L. Chan K. M. (2008). Low-intensity pulsed ultrasound on tendon healing: a study of the effect of treatment duration and treatment initiation. Am. J. Sports Med. 36, 1742–1749. 10.1177/0363546508318193 18645043

[B19] Galdiero M. Auriemma R. S. Pivonello R. Colao A. (2014). Cushing, acromegaly, GH deficiency and tendons. Muscles Ligaments Tendons J. 4, 329–332. 10.11138/mltj/2014.4.3.329 25489551 PMC4241424

[B20] Galloway M. T. Lalley A. L. Shearn J. T. (2013). The role of mechanical loading in tendon development, maintenance, injury and repair. J. Bone Joint Surg. Am. 95, 1620–1628. 10.2106/JBJS.l.01004 24005204 PMC3748997

[B200] Gibson W. (1998). Are “spontaneous” Achilles tendon ruptures truly spontaneous? Br. J. Sports Med. 32:266. 9773186

[B21] Hast M. W. Abboud J. A. Soslowsky L. J. (2014). Exploring the role of hypercholesterolemia in tendon health and repair. Muscles Ligaments Tendons J. 4, 275–279. 10.11138/mltj/2014.4.3.275 25489542 PMC4241415

[B22] Holmes G. B. Lin J. (2006). Etiologic factors associated with symptomatic achilles tendinopathy. Foot Ankle Int. 27, 952–959. 10.1016/0021-9290(94)90155-4 17144959

[B23] Jamali A. A. Afshar P. Abrams R. A. Lieber R. L. (2000). Skeletal muscle response to tenotomy. Muscle Nerve 23, 851–862. 10.1002/(sici)1097-4598(200006)23:6<851::aid-mus3>3.0.co;2-a 10842260

[B24] Kannas T. M. Amiridis I. G. Arabatzi F. Katis A. Kellis E. (2015). Changes in specific jumping performance after detraining period. J. Sports Med. Phys. Fitness 55, 1150–1156. 25323480

[B25] Kannus P. Natri A. (1997). Etiology and pathophysiology of tendon ruptures in sports. Scand. J. Med. Sci. Sports 7, 107–112. 10.1111/j.1600-0838.1997.tb00126.x 9211611

[B26] Kaux J. F. Forthomme B. Goff C. L. Crielaard J. M. Croisier J. L. (2011). Current opinions on tendinopathy. J. Sports Sci. Med. 10, 238–253. 24149868 PMC3761855

[B47] Kondratko-Mittnacht J. Lakes R. Vanderby R. Jr. (2015). Shear loads induce cellular damage in tendon fascicles. J. Biomech. 48, 3299–3305. 10.1016/j.jbiomech.2015.06.006 26162546 PMC5051692

[B27] Kubo K. Ikebukuro T. Maki A. Yata H. Tsunoda N. (2012). Time course of changes in the human Achilles tendon properties and metabolism during training and detraining *in vivo*. Eur. J. Appl. Physiol. 112, 2679–2691. 10.1007/s00421-011-2248-x 22105708

[B28] Kubo K. Ikebukuro T. Yata H. Tsunoda N. Kanehisa H. (2010). Effects of training on muscle and tendon in knee extensors and plantar flexors *in vivo*. J. Appl. Biomech. 26, 316–323. 20841623 10.1123/jab.26.3.316

[B29] Kubo K. Ishida Y. Komuro T. Tsunoda N. Kanehisa H. Fukunaga T. (2007). Age-related differences in the force generation capabilities and tendon extensibilities of knee extensors and plantar flexors in men. J. Gerontol. A Biol. Sci. Med. Sci. 62, 1252–1258. 10.1093/gerona/62.11.1252 18000145

[B290] Leigh D. R. Abreu E. L. Derwin K. A. (2008). Changes in gene expression of individual matrix metalloproteinases differ in response to mechanical unloading of tendon fascicles in explant culture. J. Orthop. Res. 26, 1306–1312. 10.1002/jor.20650 18404723 PMC6100787

[B30] Lui P. P. Maffulli N. Rolf C. Smith R. K. (2011). What are the validated animal models for tendinopathy? Scand. J. Med. Sci. Sports 21, 3–17. 10.1111/j.1600-0838.2010.01164.x 20673247

[B31] Maeda E. Ohashi T. (2015). Mechano-regulation of gap junction communications between tendon cells is dependent on the magnitude of tensile strain. Biochem. Biophys. Res. Commun. 465, 281–286. 10.1016/j.bbrc.2015.08.021 26260322

[B32] Maffulli N. Longo U. G. Loppini M. Denaro V. (2010). Current treatment options for tendinopathy. Expert Opin. Pharmacother. 11, 2177–2186. 10.1517/14656566.2010.495715 20569088

[B33] Maffulli N. Longo U. G. Maffulli G. D. Rabitti C. Khanna A. Denaro V. (2011). Marked pathological changes proximal and distal to the site of rupture in acute Achilles tendon ruptures. Knee Surg. Sports Traumatol. Arthrosc. 19, 680–687. 10.1007/s00167-010-1193-2 20563556

[B34] Maffulli N. Binfield P. M. King J. B. (1998). Tendon problems in athletic individuals. J. Bone Joint Surg. Am. 80, 142–144. 9469319

[B35] Maffulli N. Wong J. Almekinders L. C. (2003). Types and epidemiology of tendinopathy. Clin. Sports Med. 22, 675–692. 10.1016/s0278-5919(03)00004-8 14560540

[B36] Magnusson S. P. Langberg H. Kjaer M. (2010). The pathogenesis of tendinopathy: balancing the response to loading. Nat. Rev. Rheumatol. 6, 262–268. 10.1038/nrrheum.2010.43 20308995

[B37] Malliaras P. Kamal B. Nowell A. Farley T. Dhamu H. Simpson V.. (2013). Patellar tendon adaptation in relation to load-intensity and contraction type. J. Biomech. 46, 1893–1899. 10.1016/j.jbiomech.2013.04.022 23773532

[B38] McMahon G. E. Morse C. I. Burden A. Winwood K. Onambélé-Pearson G. L. (2013). The manipulation of strain, when stress is controlled, modulates *in vivo* tendon mechanical properties but not systemic TGF-β1 levels. Physiol. Rep. 1:e00091. 10.1002/phy2.91 24303165 PMC3841029

[B39] Moerch L. Pingel J. Boesen M. Kjaer M. Langberg H. (2013). The effect of acute exercise on collagen turnover in human tendons: influence of prior immobilization period. Eur. J. Appl. Physiol. 113, 449–455. 10.1007/s00421-012-2450-5 22790487

[B390] Nakama L. H. King K. B. Abrahamsson S. Rempel D. M. (2005). Evidence of tendon microtears due to cyclical loading in an in vivo tendinopathy model. J. Orthop. Res. 23, 1199–1205. 10.1016/j.orthres.2005.03.006 16140201

[B40] Oliva F. Misiti S. Maffulli N. (2014a). Metabolic diseases and tendinopathies: the missing link. Muscles Ligaments Tendons J. 4, 273–274. 25489541 PMC4241414

[B41] Oliva F. Osti L. Padulo J. Maffulli N. (2014b). Epidemiology of the rotator cuff tears: a new incidence related to thyroid disease. Muscles Ligaments Tendons J. 4, 309–314. 10.11138/mltj/2014.4.3.309 25489548 PMC4241421

[B42] Rees J. D. Wilson A. M. Wolman R. L. (2006). Current concepts in the management of tendon disorders. Rheumatology (Oxford) 45, 508–521. 10.1093/rheumatology/kel046 16490749

[B43] Riley G. (2004). The pathogenesis of tendinopathy. A molecular perspective. Rheumatology (Oxford) 43, 131–142. 10.1093/rheumatology/keg448 12867575

[B44] Salamanna F. Frizziero A. Pagani S. Giavaresi G. Curzi D. Falcieri E.. (2015). Metabolic and cytoprotective effects of *in vivo* peri-patellar hyaluronic acid injections in cultured tenocytes. Connect. Tissue Res. 56, 35–43. 10.3109/03008207.2014.979166 25333747

[B440] Sandberg O. H. Dånmark I. Eliasson P. Aspenberg P. (2015). Influence of a lower leg brace on traction force in healthy and ruptured Achilles tendons. Muscles Ligaments Tendons J. 5, 63–67. 10.11138/mltj/2015.5.2.063 26261783 PMC4496020

[B45] Sandri M. (2008). Signaling in muscle atrophy and hypertrophy. Physiology (Bethesda) 23, 160–170. 10.1152/physiol.00041.2007 18556469

[B46] Sharma P. Maffulli N. (2005). Basic biology of tendon injury and healing. Surgeon 3, 309–316. 10.1016/s1479-666x(05)80109-x 16245649

[B49] Snedeker J. G. Gautieri A. (2014). The role of collagen crosslinks in ageing and diabetes - the good, the bad and the ugly. Muscles Ligaments Tendons J. 4, 303–308. 10.11138/mltj/2014.4.3.303 25489547 PMC4241420

[B50] Stafilidis S. Arampatzis A. (2007). Muscle - tendon unit mechanical and morphological properties and sprint performance. J. Sports Sci. 25, 1035–1046. 10.1080/02640410600951589 17497405

[B51] Stanley R. L. Goodship A. E. Edwards B. Firth E. C. Patterson-Kane J. C. (2008). Effects of exercise on tenocyte cellularity and tenocyte nuclear morphology in immature and mature equine digital tendons. Equine Vet. J. 40, 141–146. 10.2746/042516408x266097 18093891

[B52] Torricelli P. Fini M. Giavaresi G. Carpi A. Nicolini A. Giardino R. (2006). Effects of systemic glucocorticoid administration on tenocytes. Biomed. Pharmacother. 60, 380–385. 10.1016/j.biopha.2006.07.003 16928425

[B53] Torricelli P. Veronesi F. Pagani S. Maffulli N. Masiero S. Frizziero A.. (2013). *In vitro* tenocyte metabolism in aging and oestrogen deficiency. Age (Dordr.) 35, 2125–2136. 10.1007/s11357-012-9500-0 23274854 PMC3825001

[B54] Urwin M. Symmons D. Allison T. Brammah T. Busby H. Roxby M.. (1998). Estimating the burden of musculoskeletal disorders in the community: the comparative prevalence of symptoms at different anatomical sites and the relation to social deprivation. Ann. Rheum. Dis. 57, 649–655. 10.1136/ard.57.11.649 9924205 PMC1752494

[B55] Warden S. J. (2009). Development and use of animal models to advance tendinopathy research. Front. Biosci. (Landmark Ed.) 14, 4588–4597. 10.2741/3551 19273373

[B56] Zhang Q. Joshi S. K. Manzano G. Lovett D. H. Kim H. T. Liu X. (2013). Muscle extracellular matrix degradation and contractibility following tendon rupture and disuse. Muscles Ligaments Tendons J. 3, 35–41. 10.11138/mltj/2013.3.1.035 23885343 PMC3676162

